# Uncovering the hidden: complexity and strategies for diagnosing latent tuberculosis

**DOI:** 10.15698/mic2017.11.596

**Published:** 2017-10-24

**Authors:** Mario Alberto Flores-Valdez

**Affiliations:** 1 Centro de Investigación y Asistencia en Tecnología y diseño del Estado de Jalisco, A.C., Biotecnología Médica y Farmacéutica, Av. Normalistas 800, Col. Colinas de la Normal, Guadalajara, Jalisco, Mexico, 44270.

**Keywords:** tuberculosis, latent infection, diagnosis, proteomics, transcriptomics, point-of-care, immune response

## Abstract

Tuberculosis produces two clinical manifestations: active and latent (non-apparent) disease. The latter is estimated to affect one-third of the world population and constitutes a source of continued transmission should the disease emerge from its hidden state (reactivation). Methods to diagnose latent TB have been evolving and aim to detect the disease in people who are truly infected with *M. tuberculosis*, versus those where other mycobacteria, or even other pathologies not related to TB, are present. The current use of proteomic and transcriptomic approaches may lead to improved detection methods in the coming years.

Tuberculosis (TB) is a bacterial infection that mostly affects the lungs and is produced by members of the *Mycobacterium tuberculosis* (Mtb) complex. For people who come into contact with these bacteria, there can be three main outcomes: (i) elimination of the pathogen by their defenses (immune system), which is calculated to occur in 90% of the cases, (ii) containment of the bacteria, which are not eliminated but cannot cause disease either, named latent TB, and (iii) active disease, where affected people show visible signs of being sick. According to the World Health Organization (WHO), an estimated 2 billion people are latently infected with TB 1.

Diagnosing active disease is relative simple, given that patients manifest signs and symptoms such as coughing, weight loss, fever, and the capacity to infect others, due to the presence of bacteria in a mixture of saliva and mucus that is coughed up (sputum). Conversely, latent TB remains hidden until a condition allows the bacteria to break the immunological balance in their favor and continue propagating to new people. Therefore, a number of strategies have been developed by scientists around the world to try to detect TB in apparently healthy people.

Perhaps one of the most widely used tools to diagnose latent TB is the Tuberculin Skin Test (TST). In this method, a complex protein mixture termed Purified Protein Derivative (PPD) is applied to the forearm of a person suspected to be infected. This might or might not lead to a localized inflammatory reaction of variable size 2. The main drawback of this test is that PPD shares many of the same components that are present in the vaccine used to prevent disseminated TB in children (*Mycobacterium bovis* BCG) and other bacteria living in the environment that cause no disease to humans. Because of this, new strategies that take into account the differences between components (antigens) present in bacteria capable of producing disease, and those present in the vaccine, are currently used to diagnose latent TB infection. In one such strategy, a blood sample taken from the patient is mixed with these antigens, and a specific defense component is measured (interferon gamma; therefore, these are known as interferon gamma release assays - IGRAs). Analysis is performed in the lab, so that the inflammatory reaction takes place out of the body, thus causing no further discomfort to the patient, beyond that of the primary blood extraction. As research has continued, the number and identity of antigens has varied, which allows doctors to be more confident of the results. Both TST and IGRAs are relatively simple to perform and do not require highly sophisticated equipment.

In another method based on blood samples, cells are separated from the proteinaceous liquid (serum), and the serum is used as a source of small defense molecules (antibodies) capable of detecting components present in pathogens. Typically, these antibodies are later recognized by another antibody that was modified in the lab, to allow detection by color, light, or fluorescence emission, in assays known as Western blot, ELISA, or lateral-flow tests (such as those used for pregnancy tests, also known as point-of-care devices). These assays rely on the presence of different antibodies recognizing distinct antigens, depending on the status of disease 3. These tests also require moderately sophisticated equipment and trained personnel, with the exception of lateral-flow assays.

These previously mentioned assays, however, can still lead to false positive or false negative results. Another strategy using blood samples, takes advantage of the current capacity to separate, label, and identify, single cells (or even minuscule components from their interior, such as nucleic acids - DNA or RNA), some of which are defense system cells that have been proposed to be markers of the status of infection 4. Assays like these require highly sophisticated equipment and highly trained personnel.

Finally, the source of bacterial antigens has long been dependent on different ways of culturing them in the lab. This has led to a wide variety of antigens assayed separately, sometimes repeatedly, by different investigators. In some cases, conditions mimicking active disease were used, while in others, conditions thought to occur during latent TB were employed. Recently, a culture-independent assay has been developed, which allows the simultaneous scanning of the whole set of antigenic proteins. This has the potential to encompass all the antigenic proteins that Mtb could use at different phases of infection, and it has been used to discriminate TBs status depending on location of patients and/or co-infection or not with Human Immunodeficiency Virus (HIV) 5. It seems reasonable that this technology could also be applied to study latent TB cases and determine if a highly specific test can be developed. A schematic representation of the various types of methods employed to diagnose latent TB infection is shown in Figure 1.

**Figure 1:**
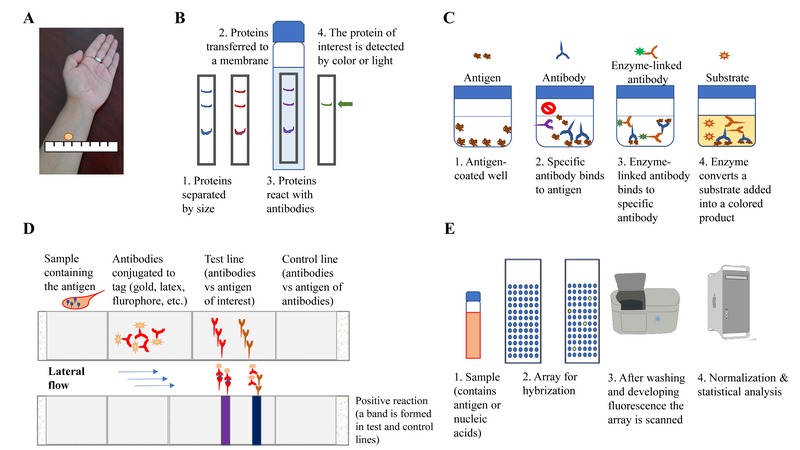
FIGURE 1: Schematic representation of how diagnostic methods have evolved to detect TB infection. **(A)** The Tuberculin Skin Test (also known as Mantoux test) depends on the inflammatory response, mediated by immune cells influx, localized at the site of injection in the forearm; **(B)** detection of antigenic proteins by a technique called “Western blot”, where antibodies are present in the blood of a suspected TB-infected person, and detect antigens present in a membrane; **(C)** another approach that detects antigens via antibodies is the “ELISA” test; this is used, for example in the Interferon gamma release assays. The difference between “Western blot” and “ELISA” assays is where the reaction takes places, in a matrix or in solution, respectively; **(D)** a variation of “Western blot” is the “Lateral flow assay”, where multiple antigen-antibody reaction occurs simultaneously in a membrane, and these are detected as colored bands; **(E)** finally, the whole set of protein or nucleic acids sequences can be screened via hybridization and using fluorescent labels. Considering the vast number of reactions occurring here, normalization and statistical analyses are conducted to ascertain what signal is real and not background/noise and therefore not relevant.

In summary, diagnosis of latent TB has evolved. Hopefully in the short term, a non-invasive, highly sensitive and specific test will be available so that patients can be treated with better chances of eliminating their persistently hidden Mtb.

## References

[1] World Health Organization (2016) Global tuberculosis report 2016.. http://www.who.int/tb/publications/global_report/en/.

[2] Center for Disease Control and Prevention Tuberculin Skin Testing.. https://www.cdc.gov/tb/publications/factsheets/testing/skintesting.htm.

[3] Castro-Garza J, Garcia-Jacobo P, Rivera-Morales LG, Quinn FD, Barber J, Karls R, Haas D, Helms S, Gupta T, Blumberg H, Tapia J, Luna-Cruz I, Rendon A, Vargas-Villarreal J, Vera-Cabrera L, Rodriguez-Padilla C (2017). Detection of anti-HspX antibodies and HspX protein in patient sera for the identification of recent latent infection by Mycobacterium tuberculosis.. PLoS One.

[4] Pathakumari B, Devasundaram S, Raja A (2017). Altered expression of antigen-specific memory and regulatory T cell subsets differentiate latent and active Tuberculosis.. Immunology.

[5] Song LS, Wallstrom G, Yu XB, Hopper M, Van Duine J, Steel J, Park J, Wiktor P, Kahn P, Brunner A, Wilson D, Jenny-Avital ER, Qiu J, Labaer J, Magee DM, Achkar JM (2017). Identification of Antibody Targets for Tuberculosis Serology using High-Density Nucleic Acid Programmable Protein Arrays.. Mol Cell Proteomics.

